# National trends and survival outcomes of penile squamous cell carcinoma based on human papillomavirus status

**DOI:** 10.1002/cam4.4258

**Published:** 2021-10-10

**Authors:** Juan Chipollini, Grant Pollock, Chiu‐Hsieh Hsu, Ken Batai, Alejandro Recio‐Boiles, Benjamin R. Lee

**Affiliations:** ^1^ Department of Urology University of Arizona Tucson Arizona USA; ^2^ Department of Epidemiology and Biostatistics University of Arizona Tucson Arizona USA; ^3^ Department of Medicine University of Arizona Cancer Center Tucson Arizona USA

**Keywords:** clinical observations, epidemiology, survival, viral infection

## Abstract

**Background:**

There are no series evaluating penile squamous cell carcinoma (pSCC) based on human papillomavirus (HPV) infection. Herein, we present national registry data on clinical and survival outcomes for pSCC based on HPV status.

**Methods:**

We performed a retrospective review of 1224 pSCC patients with known HPV staining from the National Cancer Database. Patients with cM1 disease, those who did not receive treatment, or had missing follow‐up data were excluded. Logistic regression identified factors associated with locally aggressive disease. Univariable, multivariable, and inverse probability of treatment weighting (IPTW)‐Cox proportional hazard modeling were used to assess hazard ratios (HR) associated with overall survival (OS).

**Results:**

After exclusion criteria, we identified 825 cases of which 321 (38.9%) were HPV positive. The HPV‐positivity rate did not significantly change by year. HPV‐positive patients were younger, had lower Charlson‐Deyo performance score, and resided in areas with both lower median household income and lower school education completion. HPV‐positive tumors presented with lower American Joint Committee on Cancer clinical T‐stage (cT), poorer differentiation, lower rates of lymphovascular invasion (LVI), but more node‐positive disease (cN+). For those who underwent lymph node surgery, there were no differences in final pathologic stage, upstaging, or presence of extranodal extension. Only tumor differentiation, LVI, and performance score were independent predictors for locally aggressive disease. HPV status was not a predictor of OS (IPTW‐HR:0.89, *p* = 0.13).

**Conclusions:**

In the largest series evaluating pSCC based on HPV status, HPV‐positive tumors were associated with lower cT stages, less LVI, but more cN + disease. More studies on prognostic factors are needed, and time may still be immature to use HPV information for risk stratification.

## Graphical abstract

Infection with human papillomavirus (HPV) is endemic and a causative agent for several malignancies in both male and female populations. In this study, we queried a large, cancer registry to look for clinical and survival differences between HPV‐positive versus negative penile tumors. Our results noted a lack of prognostic impact for HPV‐derived tumors, although more studies will be required to validate results.

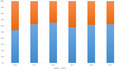

## 1 INTRODUCTION

Squamous cell carcinoma of the penis (pSCC) is a rare malignancy in Western nations.^1^, ^2^, ^3^, ^4^ The overall incidence in the United States (US) is approximately 0.69 per 100,000 men and occurrence is associated with increasing age at diagnosis.^5^ The etiology of pSCC is multifactorial with well‐recognized risk factors, including phimosis, smoking, chronic irritation, socioeconomic status, immune response, and human papillomavirus (HPV) infection.^4^, ^6^, ^7^ Current epidemiologic factors and molecular pathways for pSCC continue to be investigated although disease‐specific survival continues to be poor to this date.

Although there have been advances in the understanding of penile carcinogenesis and tumor microenvironment,^8^, ^9^ the unique molecular mechanisms underlying pSCC remain poorly understood. At present, tumors are thought to arise from progression of precursor lesions arising from separate HPV‐dependent and HPV‐independent pathways.^9^, ^10^, ^11^ While penile intraepithelial neoplasia (PeIN) is thought to be the precursor of SCC,^12^ only a small portion develop into invasive tumors with currently no established prognostic biomarkers identified to date.

Correct etiologic classification of penile lesions during diagnostic work‐up has the potential to allow for individual management and therapeutic decisions. The few published series worldwide have shown mixed results in survival of penile and other HPV‐related cancers when compared to HPV‐independent cohorts.^13^, ^14^, ^15^, ^16^, ^17^ In the US, there have been no large studies evaluating prognostic differences of pSCC patients based on HPV status. We reviewed the proportion of HPV‐derived penile tumors and their association with survival outcomes using the largest US‐cancer registry cohort to date.

## 2 MATERIALS AND METHODS

### 2.1 Data source

The National Cancer Database (NCDB) is a hospital‐based cancer registry that collects high quality, de‐identified, and internally appraised cancer data from more than 1500 US hospitals approved by the American College of Surgeons and the Commission on Cancer.^18^ The University of Arizona review board approval was not required for this study because the dataset is publicly available and de‐identified.

### 2.2 Study population

**FIGURE 1:**
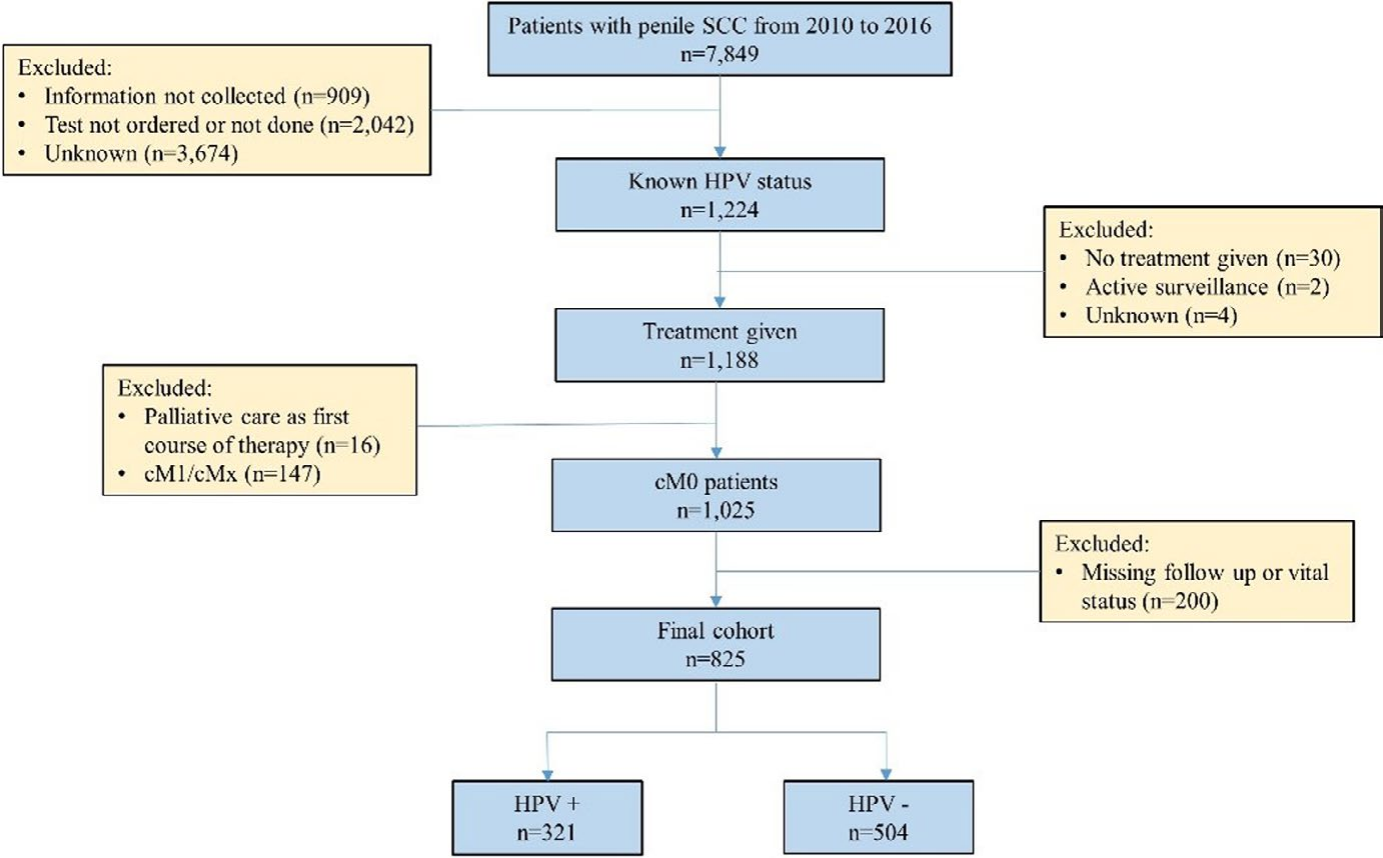
Flow diagram detailing patient inclusion and exclusion for determining the analysis group

We performed a retrospective review of the NCDB for adults with previously untreated pSCC diagnosed between 2010 and 2015. HPV status was first recorded to NCDB coding in 2010. Squamous cell carcinoma was identified with International Classification of Diseases for Oncology, Third Edition (ICD‐O‐3) histologic codes 8050–8084. ICD‐O‐3 topographical codes in our cohort included C60.0‐C60.2 and C60.8‐C60.9. Patients with documented HPV (HPV ‐ 16, HPV ‐ 18, or high‐risk HPV, NOS) were considered positive for HPV. Patients who did not receive treatment, those with cM1 disease, patients with missing follow‐up or vital status, and those who were treated with palliative intent were excluded from analysis (Figure 1).

### 2.3 Study variables

Covariates for the analysis included the following: treatment year, age, race, ethnicity, Charlson‐Deyo performance score, insurance status, treating facility type, hospital geographic region, and median income and education status by patient zip code. Clinical and pathologic staging data included tumor stage, differentiation, type of primary site surgery, surgical margin status, receipt of regional lymph node (LN) surgery, and presence of extranodal extension (ENE) or lymphovascular invasion (LVI). Only 16% of patients underwent LN dissection providing pN status only for that group. We used the clinical and pathologic staging defined in the seventh edition of the American Joint Committee on Cancer (AJCC) Staging Manual.^19^

### 2.4 Statistical analysis

The primary end point was overall survival (OS) from the initial diagnosis to the date of death or censoring at the last follow‐up. Descriptive statistics for clinical and socio‐demographic characteristics were compared based on HPV status. Differences in proportions were derived using two‐sample *t*‐test for continuous variables and Fisher's exact test for categorical variables. Univariable and multivariable logistic regression was used to determine factors for aggressive disease defined as cT3‐4 and/or N+ disease. For the survival analysis, we performed univariable and multivariable Cox regression to determine the hazard ratio (HR) of OS between HPV‐positive versus negative patients. The model accounted for differences in age, race, income, education, tumor size, grade, margin, type of primary site surgery, and receipt of LN surgery. In addition, we used inverse probability of treatment weighting (IPTW) in order to balance covariates and account for treatment differences between HPV‐positive and negative patients in our survival model.^20^ All statistical analyses were conducted using SAS version 9.4 (SAS Institute Inc.).

## 3 RESULTS

### 3.1 Patient characteristics

A total of 825 cases were identified of which 321 (38.9%) were HPV positive (HPV+). The median patient age was 64 years (interquartile range [IQR], 53–74). The median follow‐up from diagnosis to last examination or death was 29.9 months (14.8–48.5). The demographic variables and the results of univariable analysis of the patient characteristics are listed in Table 1. HPV + patients were younger, and resided in areas with lower median household income and lower high school education completion.

**TABLE 1:** Characteristics of overall cohort and factors associated with human papillomavirus status

Variables	HPV		*p*‐value
*N* (%) or median (IQR)	Negative	Positive	
Patients	504 (61.1)	321 (38.9)	
Age (year)	64.77±14.86	60.08±15.13	**<0.01**
Race			**<0.01**
White	421 (83.5)	254 (79.1)	
Black	45 (8.9)	52 (16.2)	
Other	32 (6.3)	11 (3.4)	
Unknown	6 (1.2)	4 (1.2)	
Ethnicity			0.27
Non‐Hispanic	441 (87.5)	268 (83.5)	
Hispanic	52 (10.3)	44 (13.7)	
Unknown	11 (2.2)	9 (2.8)	
Charlson‐Deyo score			0.56
0	352 (69.8)	219 (68.2)	
1	104 (20.6)	64 (19.9)	
>1	48 (9.5)	38 (11.8)	
Median household income by zip code			**0.01**
<$38,000	89 (17.7)	88 (27.5)	
$38,000–47,999	142 (28.2)	80 (25)	
$48,000–62,999	128 (25.4)	73 (22.8)	
$63,000 or more	144 (28.6)	79 (24.7)	
Facility type			0.99
Academic/research program	249 (52.4)	153 (52.4)	
Community/comprehensive/other	226 (47.6)	139 (47.6)	
Rurality			0.88
Metropolitan	369 (76.6)	247 (77.9)	
Suburban	61 (12.7)	39 (12.3)	
Rural	52 (10.8)	31 (9.8)	
Insurance			0.24
None	28 (5.6)	10 (3.1)	
Private insurance	168 (33.3)	114 (35.5)	
Medicare	243 (48.2)	143 (44.5)	
Medicaid/Other government	54 (10.7)	45 (14)	
Unknown	11 (2.2)	9 (2.8)	
No high school degree by zip code			**0.03**
21% or more	89 (17.7)	76 (23.8)	
13%–20.9%	151 (30)	81 (25.3)	
7%–12.9%	144 (28.6)	105 (32.8)	
<7%	119 (23.7)	58 (18.1)	

Bold values indicate statistical significance.

Abbreviations: HPV, human papillomavirus.

### 3.2 Tumor characteristics

**FIGURE 2:**
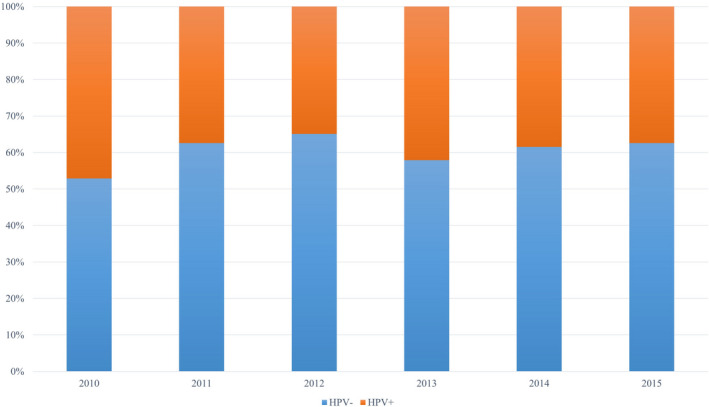
Proportion of human papillomavirus positive and negative tumors by year

HPV positivity non‐significantly decreased from 47.1% in 2010 to 37.4% in 2015 (Cochran‐Armitage *p* = 0.56). (Figure 2). HPV + tumors presented with lower clinical T‐stage (cT), poorer differentiation, and were less likely to demonstrate LVI. Patient with HPV + tumors also presented with more palpable adenopathy although not statistically significant. (Table 2).

**TABLE 2:** Clinical and postoperative outcomes based on human papillomavirus status

Variables	HPV		*p*‐value
N (%)	Negative	Positive	
cT stage			**<0.01**
T0/Ta/Tis	109 (21.6)	106 (33)	
T1	189 (37.5)	82 (25.5)	
T2	58 (11.5)	36 (11.2)	
T3/T4	41 (8.1)	19 (5.9)	
Tx	107 (21.2)	78 (24.3)	
cN stage			0.75
N0	417 (83.2)	257 (80.8)	
N1	15 (3)	12 (3.8)	
N2	22 (4.4)	20 (6.3)	
N3	14 (2.8)	8 (2.5)	
Nx	33 (6.6)	21 (6.6)	
Tumor grade			**<0.01**
Well differentiated	123 (24.4)	51 (15.9)	
Moderately differentiated	172 (34.1)	78 (24.3)	
Poorly differentiated	69 (13.7)	48 (15)	
Undifferentiated/not applicable	140 (27.8)	144 (44.9)	
Primary site surgery			**0.02**
Local tumor treatment	199 (39.5)	165 (51.4)	
Partial penectomy	209 (41.5)	109 (34)	
Total/radical penectomy	77 (15.3)	30 (9.3)	
Surgery, NOS	3 (0.6)	1 (0.3)	
Regional lymph node surgery	84 (16.7)	48 (15)	**0.04**
Lymphovascular invasion	56 (11.1)	35 (10.9)	**0.03**
Surgical margin positive	56 (11.1)	41(12.8)	**<0.01**

Bold values indicate statistical significance.

Abbreviations: cN, clinical node stage; cT, clinical tumor stage; HPV, human papillomavirus.

### 3.3 Lymphatic characteristics

Approximately 16% of patients underwent regional lymph node dissection (LND). There were no differences in the median number of LNs examined: 17 (8–31) versus 16.5 (7.3–26.5) for HPV‐negative versus positive patients, respectively (*p* = 0.63). No differences were found in final pN stage, clinical to pathologic nodal upstaging, or presence of ENE (Table 3). Only 15 patients received postoperative radiation with no impact on survival.

**TABLE 3:** Outcomes after lymph node surgery (*n* = 132)

Variables	HPV		*p*‐value
*N* (%)	Negative	Positive	
pN stage			0.23
pN0	38 (46.9)	22 (46.8)	
pN1	6 (7.4)	8 (17)	
pN2	14 (17.3)	10 (21.3)	
pN3	16 (19.8)	6 (12.8)	
pNx	7 (8.6)	1 (2.1)	
Extranodal extension	16 (19)	6 (12.5)	0.57
Postoperative radiation	10 (11.9)	5 (10.4)	0.74
Median number of nodes (IQR)	17 (8–31)	16.5 (7.3–26.5)	0.63

Abbreviations: HPV, human papillomavirus; IQR, interquartile range; pN, pathologic node stage.

### 3.4 HPV not found to be a predictor for aggressive disease

Independent predictors associated with locally advanced disease (cT3‐4/N+) at initial diagnosis were tumor grade (moderately differentiated: odds ratio [OR], 4.29; 95%CI, 2.15–8.56 and poorly differentiated: OR, 8.58; 95%CI, 3.97–18.6), presence of LVI (OR, 4.05; 95%CI, 1.52–4.80), and Charlson‐Deyo score of 1 (OR, 2.01; 95%CI, 1.19–3.39) (Table 4). HPV positivity was not an independent factor (OR, 0..95; 95%CI, 0.59–1.53) in multivariable analysis.

**TABLE 4:** Predictors for high‐risk (cT3‐4/N+ disease) features (*n* = 132)

	Univariable		Multivariable	
	OR (95%CI)	*p*	OR (95%CI)	*p*
Age	1.00 (0.99–1.02)	0.56	0.99 (0.98–1.01)	0.34
Tumor grade (Ref: well differentiated)				
Moderately differentiated	4.46 (2.29–8.67)	**<0.01**	4.29 (2.15–8.56)	**<0.01**
Poorly differentiated	8.90 (4.28–18.5)	**<0.01**	8.58 (3.97–18.6)	**<0.01**
Undifferentiated/Anaplastic	0.64 (0.29–1.42)	0.27	0.58 (0.25–1.35)	0.206
LVI (Ref: not present)				
Present	6.54 (3.82–11.2)	**<0.01**	4.05 (2.24–7.33)	**<0.01**
Unknown	1.52 (0.94–2.45)	0.09	2.71 (1.52–4.80)	**<0.01**
Ethnicity (Ref: non‐Hispanic)				
Hispanic	1.06 (0.60–1.89)	0.84	1.76 (0.91–3.38)	0.09
Unknown	0.48 (0.11–2.10)	0.33	0.83 (0.30–11.7)	0.72
Race (Ref: White)				
Black	1.55 (0.89–2.68)	0.12	1.76 (0.91–3.38)	0.09
Other	0.83 (0.33–2.04)	0.68	0.83 (0.30–2.33)	0.88
Charlson‐Deyo score (Ref: 0)				
1	1.83 (1.17–2.86)	**<0.01**	2.01 (1.19–3.39)	**<0.01**
2	0.78 (0.32–1.92)	0.59	0.60 (0.23–1.59)	0.30
≥3	1.48 (0.57–3.83)	0.42	1.39 (0.43–4.49)	0.59
HPV positivity	0.98 (0.66–1.45)	0.90	0.95 (0.59–1.53)	0.83

Bold values indicate statistical significance.

Abbreviations: CI, confidence interval; HPV, human papillomavirus; LVI, lymphovascular invasion; OR, odds ratio; Ref, reference.

### 3.5 HPV not found to be a predictor for overall survival

HPV was not a significant factor in both univariable (HR, 0.85; 95%CI, 0.65–1.11) and multivariable (HR, 0.89; 95%CI, 0.67–1.19) Cox regression models. In the IPTW‐derived regression model, HPV was not a significant factor (HR, 0.89; 95%CI, 0.77–1.03) in OS (Table 5).

**TABLE 5:** Mortality risks by human papillomavirus status

HPV	Number of dead (%)	Unadjusted HR^a^ (95% CI); *p*	Adjusted^b^ HR (95% CI); *p*	IPTW^c^ HR (95% CI); *p*
Negative	149 (29.56%)	Ref	Ref	Ref
Positive	83 (25.86%)	0.85 (0.65–1.11); *p* = 0.22	0.89 (0.67–1.19); *p* = 0.43	0.89 (0.77–1.03); *p* = 0.13

^a^ Derived from Cox regression.

^b^ Adjusted for Age, Race, Income, Education, Tumor Size, Grade, Margin, Tumor stage, Primary site surgery, and Lymph node removal surgery.

^c^ IPTW derived logistic regression for HPV status with Age, Race, Income, Education, Tumor Size, Grade, Margin, Tumor stage, Primary Site Surgery, and Lymph node removal surgery as the covariates.

Abbreviations: CI, confidence interval; HPV, human papillomavirus; HR, Hazard ratio; IPTW, inverse probability of treatment weighting.

## 4 DISCUSSION

Penile carcinoma is a rare malignancy with SCC accounting for approximately 95% of tumors.^21^ Patterns of lymphatic dissemination are well established with progression and treatment of local tumors having significant physical consequences. Although previous studies have focused on the incidence of HPV‐associated cancers, very few have examined the impact of HPV on survival, and with most studies focusing on more prevalent malignancies, such as cervical and oropharyngeal cancers.^17^, ^22^ In the 6‐year window of our study, we did not see an appreciable change over time on the HPV‐positivity rate. In addition, HPV infection was not associated with more locally advanced features nor was it associated with detrimental survival outcomes.

While few population‐based case‐control studies have assessed the epidemiologic factors of penile tumors, our data are within the range of reported rates of HPV in 40%–50% of cases arising through epithelial transformations caused by HPV.^10^, ^23^, ^24^, ^25^ In our study, most HPV + tumors presented with lower cT stage and lower rates of LVI but paradoxically were also found to have poorer differentiation and more palpable adenopathy. Our study showed HPV status to have only etiologic implications with no significant prognostic corollary. Using our results, the addition of HPV subclassification of primary tumors would not add further risk stratification information into our current AJCC staging system, given its lack of prognostic applications for disease management. Nevertheless, the US population may not provide an ideal cohort to provide such conclusions and robust studies from more informative geographical areas with higher incidence of penile cancer could deliver more granular data for our current understanding of pSCC biology, prognosis, and treatment.

HPV is a well‐known risk factor for the development of cancers affecting the head and neck, cervix, anal canal, vulva, and penis.^7^, ^17^, ^26^ The molecular pathogenesis of HPV + penile tumors is thought to be similar to that of cervical cancer, for which infection with mucosal high‐risk HPV genotypes is required.^6^, ^7^, ^10^, ^15^ Infection causes expression of oncoproteins E6 and E7, which bind and inactivate tumor suppressors p53 and Rb respectively; and interfere with control of cell division and apoptosis.^11^, ^27^, ^28^ Although the rates of HPV infections have been equally common in the cervix as in the penis,^29^ HPV‐associated cervical cancer rates have been much greater than male counterparts.^16^, ^30^ Nonetheless, given the success of the quadrivalent vaccine against HPV (types 6, 11, 16, and 18) on leading substantial decreases in HPV‐associated pre‐malignant lesions in both young and older women,^31^, ^32^ an argument for promoting vaccination, along with other educational and preventative strategies, may help decrease risk of likewise pre‐malignant lesions for the male population.

Development of HPV‐negative tumors is less well understood, but has been linked to p53 mutations, similar to vulvar carcinogenesis.^11^ These tumors arise from precursor PeIN lesions, usually in chronic inflammatory settings, such as lichen sclerosus or lichen planus.^11^, ^33^ Inflammatory cells produce reactive oxygen/nitrogen species which are involved in the development and progression of several human cancers.^34^ Other markers, such as programmed death ligand 1 (PD‐L1), have been found mostly in HPV‐negative tumors.^8^, ^35^ Unlike HPV + tumors, these lack p16INK4A as a surrogate immunohistochemistry marker.^9^, ^11^, ^36^ When considering all the available evidence, it is apparent HPV‐dependent and independent tumors arise from different molecular pathways, but genetic alterations from both lead to the disruption of related tumor‐suppressing pathways. Ongoing phase II trials are underway using targeted therapy in rare solid tumors, including PD‐L1 + pSCC.

The most important predictor of pSCC survival is the extent of lymph node metastases.^25^ As there are few effective therapies when regional disease is present, surgical resection remains the cornerstone of treatment for both primary tumors and lymphatic metastasis. In our subset of patients receiving LND, we found no differences in pN stage, rate of clinical upstaging, or presence of ENE based on HPV status. However, given the few number of patients and lack of high‐risk features as well as the lack of clinically relevant information inherent to registry studies, such as HPV assessment, extent and type of LND, cancer‐specific survival, and use of perioperative therapies, our conclusions should be taken with caution until larger, prospective studies can be performed. In our cohort, only 15 patients received adjuvant radiation therapy after LND so no further conclusions can be drawn about this therapeutic option. So far multimodal therapy in the form of radiotherapy or chemoradiotherapy has been studied in retrospective series. One study of 51 patients found adjuvant chemoradiotherapy and HPV + status improved locoregional control for pN + patients.^37^ Another large, multicenter study showed perioperative radiation to be more effective in patients with HPV + tumors with TP53 mutation thought to enhance radiosensitivity.^38^ Prospective investigation of HPV + tumors treated with multimodal therapy is required to further delineate their roles in optimizing pSCC treatment.

Newer models of classification have separated subtypes of pSCC into HPV and non‐HPV related as they demonstrate morphological and prognostic differences.^39^ Most recently, PeIN has been classified into differentiated and undifferentiated according to HPV status. Undifferentiated/HPV‐associated PeIN can be further subdivided into basaloid, warty, and warty‐basaloid subtypes, while differentiated PeIN is characterized by involvement of the basal layers of epithelium by way of atypia, acanthosis, parakeratosis, and lichen sclerosus.^40^ Recent TNM staging for other malignancies, such as head and neck have included subclassification based on HPV positivity due to associated improved survival when compared non‐HPV cases.^41^ One study of 171 patients has suggested a survival benefit for penile cancer patients in whom HPV DNA was found in the primary tumor,^42^ while another study of 82 patients revealed that only lymphatic embolization was related to HPV status with no difference in survival rates based on HPV distinction.^43^ Our registry study of 825 cases also indicates the lack of prognostic applications for further HPV subclassification of tumors, at least until available therapies are developed to target HPV‐specific pathways with concomitant improved survival for these patients.

Although our results draw attention to the rate of HPV‐derived penile tumors in the US, our study has important limitations. Firstly, the retrospective design resulted in a notable selection bias for disease presentation owing to referral patterns which could have confounded our results. For instance, the database does not clearly detail how patients received radical surgery or regional LND, nor does it report reasons for specified therapies, functional and recurrence outcomes, or salvage treatments. Although we limited analysis to high‐risk types, due to the retrospective nature of the study, no central pathological review was available for insights into HPV serotyping. Likewise, HPV + patients were more likely to have lower stage disease, and thus perhaps a lower disease burden overall, which may potentially present bias in interpretation. However, our observational study provides generalizable data in a real‐world setting. Our retrospective study used IPTW to adjust for baseline characteristics (i.e., T‐stage) which are potential confounders while assessing the effects of HPV on mortality. While we controlled for tumor characteristics, demographics, and treatment differences, only full randomization can fully adjust for these factors. Lastly, the incidence data in the present study should not be translated directly into the national incidence because the denominator is not precisely defined within the NCDB. Nonetheless, our study has the largest cohort to date evaluating differences based on HPV status and covered a large proportion of the US population for an extremely, rare malignancy, such as pSCC.

In the era of precision medicine, there is increased interest in the use of targeted therapies given the poor responses of contemporary standard systemic therapies for advanced pSCC. Given the rarity of the disease, there continues to be immense need for multi‐institutional collaboration. The recent creation of the Global Society of Rare Genitourinary Tumors provides hope for increased support and advocacy to develop the next generation of treatments for pSCC.^44^ A better understanding of the basic biology of penile cancer can help design future prospective trials and offer insights into potential precision medicine approaches for this deadly disease.

## 5 CONCLUSION

Further classification of penile tumors according to HPV status did not correlate with disease or survival outcomes in a North American cohort of patients. Future studies evaluating HPV prevalence are necessary to assess its potential effect as an actionable target of therapy or as part of prevention programs in the male population, such as vaccination. As far as the search for prognostic factors is concerned, time is still immature to use HPV information for risk stratification and more studies are required.

## ETHICAL APPROVAL STATEMENT

Institutional board approval was not required for this study because the dataset is publicly available and de‐identified.

## CONFLICT OF INTEREST

The authors have no competing interests.

## Data Availability

The data used in the study are derived from a de‐identified database available from the American College of Surgeons.

## References

[1] Colberg C , van der Horst C , Jünemann KP , Naumann CM . Epidemiology of penile cancer. Urologe A. 2018;57:408‐412. 29468279 10.1007/s00120-018-0593-7

[2] Rippentrop JM , Joslyn SA , Konety BR . Squamous cell carcinoma of the penis: evaluation of data from the surveillance, epidemiology, and end results program. Cancer. 2004;101:1357‐1363. 15316902 10.1002/cncr.20519

[3] Persky L . Epidemiology of cancer of the penis. Recent Results Cancer Res. 1977;1:97‐109. 10.1007/978-3-642-81095-4_11 325611

[4] Douglawi A , Masterson TA . Updates on the epidemiology and risk factors for penile cancer. Transl Androl Urol. 2017;6:785‐790. 29184774 10.21037/tau.2017.05.19 PMC5673812

[5] Barnholtz‐Sloan JS , Maldonado JL , Pow‐sang J , Giuliano AR . Incidence trends in primary malignant penile cancer. Urol Oncol. 2007;25:361‐367. 17826651 10.1016/j.urolonc.2006.08.029

[6] Daling JR , Madeleine MM , Johnson LG , et al. Penile cancer: importance of circumcision, human papillomavirus and smoking in in situ and invasive disease. Int J Cancer. 2005;116:606‐616. 15825185 10.1002/ijc.21009

[7] Diorio GJ , Giuliano AR . The role of human papilloma virus in penile carcinogenesis and preneoplastic lesions: a potential target for vaccination and treatment strategies. Urol Clin North Am. 2016;43:419‐425. 27717428 10.1016/j.ucl.2016.06.003

[8] Ahmed ME , Falasiri S , Hajiran A , Chahoud J , Spiess PE . The immune microenvironment in penile cancer and rationale for immunotherapy. J Clin Med. 2020;9:3334. 10.3390/jcm9103334 PMC7603091 33080912

[9] Chipollini J , Chaing S , Azizi M , Kidd LC , Kim P , Spiess PE . Advances in understanding of penile carcinogenesis: the search for actionable targets. Int J Mol Sci. 2017;18:1777. 10.3390/ijms18081777 PMC5578166 28813024

[10] Gross G , Pfister H . Role of human papillomavirus in penile cancer, penile intraepithelial squamous cell neoplasias and in genital warts. Med Microbiol Immunol. 2004;193:35‐44. 12838415 10.1007/s00430-003-0181-2

[11] Mannweiler S , Sygulla S , Winter E , Regauer S . Two major pathways of penile carcinogenesis: HPV‐induced penile cancers overexpress p16ink4a, HPV‐negative cancers associated with dermatoses express p53, but lack p16ink4a overexpression. J Am Acad Dermatol. 2013;69:73‐81. 23474228 10.1016/j.jaad.2012.12.973

[12] Velazquez EF , Chaux A , Cubilla AL . Histologic classification of penile intraepithelial neoplasia. Semin Diagn Pathol. 2012;29:96‐102. 22641959 10.1053/j.semdp.2011.08.009

[13] Daubisse‐Marliac L , Colonna M , Trétarre B , et al. Long‐term trends in incidence and survival of penile cancer in France. Cancer Epidemiol. 2017;50:125‐131. 28898817 10.1016/j.canep.2017.08.014

[14] Eich M‐L , del Carmen Rodriguez Pena M , Schwartz L , et al. Morphology, p16, HPV, and outcomes in squamous cell carcinoma of the penis: a multi‐institutional study. Hum Pathol. 2020;96:79‐86. 31698006 10.1016/j.humpath.2019.09.013

[15] Hernandez BY , Goodman MT , Unger ER , et al. Human papillomavirus genotype prevalence in invasive penile cancers from a registry‐based United States population. Front Oncol. 2014;4:9. 24551592 10.3389/fonc.2014.00009 PMC3914298

[16] Lu Y , Li P , Luo G , Liu D , Zou H . Cancer attributable to human papillomavirus infection in China: burden and trends. Cancer. 2020;126:3719‐3732. 32484937 10.1002/cncr.32986

[17] Razzaghi H , Saraiya M , Thompson TD , Henley SJ , Viens L , Wilson R . Five‐year relative survival for human papillomavirus‐associated cancer sites. Cancer. 2018;124:203‐211. 29105738 10.1002/cncr.30947 PMC5793215

[18] Sharma P , Ashouri K , Zargar‐Shoshtari K , Luchey AM , Spiess PE . Racial and economic disparities in the treatment of penile squamous cell carcinoma: results from the National Cancer Database. Urol Oncol. 2016;34(3):122.e9‐122.e15. 10.1016/j.urolonc.2015.10.001 26547834

[19] Edge SB , Compton CC . The American Joint Committee on Cancer: the 7th edition of the AJCC cancer staging manual and the future of TNM. Ann Surg Oncol. 2010;17(6):1471‐1474. 20180029 10.1245/s10434-010-0985-4

[20] Austin PC . The use of propensity score methods with survival or time‐to‐event outcomes: reporting measures of effect similar to those used in randomized experiments. Stat Med. 2014;33:1242‐1258. 24122911 10.1002/sim.5984 PMC4285179

[21] Kroon BK , Horenblas S , Nieweg OE . Contemporary management of penile squamous cell carcinoma. J Surg Oncol. 2005;89:43‐50. 15611938 10.1002/jso.20170

[22] Viens LJ , Henley SJ , Watson M , et al. Human papillomavirus‐associated cancers ‐ United States, 2008–2012. MMWR Morb Mortal Wkly Rep. 2016;65:661‐666. 27387669 10.15585/mmwr.mm6526a1

[23] Bunker CB , Shim TN . Male genital lichen sclerosus. Indian J Dermatol. 2015;60:111‐117. 25814697 10.4103/0019-5154.152501 PMC4372901

[24] Pow‐Sang MR , Ferreira U , Pow‐Sang JM , Nardi AC , Destefano V . Epidemiology and natural history of penile cancer. Urology. 2010;76:S2‐S6. 20691882 10.1016/j.urology.2010.03.003

[25] Clark PE , Spiess PE , Agarwal N , et al. Penile cancer: clinical practice guidelines in oncology. J Natl Compr Canc Netw. 2013;11:594‐615. 23667209 10.6004/jnccn.2013.0075 PMC4042432

[26] Pizzocaro G , Algaba F , Horenblas S , et al. EAU penile cancer guidelines 2009. Eur Urol. 2010;57:1002‐1012. 20163910 10.1016/j.eururo.2010.01.039

[27] Dyson N , Howley PM , Münger K , Harlow E . The human papilloma virus‐16 E7 oncoprotein is able to bind to the retinoblastoma gene product. Science. 1989;243:934‐937. 2537532 10.1126/science.2537532

[28] Scheffner M , Werness BA , Huibregtse JM , Levine AJ , Howley PM . The E6 oncoprotein encoded by human papillomavirus types 16 and 18 promotes the degradation of p53. Cell. 1990;63:1129‐1136. 2175676 10.1016/0092-8674(90)90409-8

[29] Franceschi S , Castellsagué X , Dal Maso L , et al. Prevalence and determinants of human papillomavirus genital infection in men. Br J Cancer. 2002;86:705‐711. 11875730 10.1038/sj.bjc.6600194 PMC2375316

[30] Chaturvedi AK . Beyond cervical cancer: burden of other HPV‐related cancers among men and women. J Adolesc Health. 2010;46:S20‐S26. 20307840 10.1016/j.jadohealth.2010.01.016

[31] Arbyn M , Xu L , Simoens C , Martin‐Hirsch PPL . Prophylactic vaccination against human papillomaviruses to prevent cervical cancer and its precursors. Cochrane Database Syst Rev. 2018;5:CD009069. 29740819 10.1002/14651858.CD009069.pub3 PMC6494566

[32] The FUTURE II Study Group . Quadrivalent vaccine against human papillomavirus to prevent high‐grade cervical lesions. N Engl J Med. 2007;356:1915‐1927. 17494925 10.1056/NEJMoa061741

[33] Shabbir M , Minhas S , Muneer A . Diagnosis and management of premalignant penile lesions. Ther Adv Urol. 2011;3:151‐158. 21904571 10.1177/1756287211412657 PMC3159400

[34] Kruk J , Aboul‐Enein HY . Reactive oxygen and nitrogen species in carcinogenesis: implications of oxidative stress on the progression and development of several cancer types. Mini Rev Med Chem. 2017;17:904‐919. 28245782 10.2174/1389557517666170228115324

[35] Ottenhof SR , Djajadiningrat RS , de Jong J , Thygesen HH , Horenblas S , Jordanova ES . Expression of programmed death ligand 1 in penile cancer is of prognostic value and associated with HPV status. J Urol. 2017;197:690‐697. 27697578 10.1016/j.juro.2016.09.088

[36] Martins VDCA , Cunha IW , Figliuolo G , et al. Presence of HPV with overexpression of p16INK4a protein and EBV infection in penile cancer‐a series of cases from Brazil Amazon. PLoS One. 2020;15:e0232474. 32374757 10.1371/journal.pone.0232474 PMC7202603

[37] Yuan Z , Naghavi AO , Tang D , et al. The relationship between HPV status and chemoradiotherapy in the locoregional control of penile cancer. World J Urol. 2018;36:1431‐1440. 29589134 10.1007/s00345-018-2280-0 PMC7771321

[38] Bandini M , Ross JS , Zhu Y , et al. Association between human papillomavirus infection and outcome of perioperative nodal radiotherapy for penile carcinoma. Eur Urol Oncol. 2020;386:1–9. 10.1016/j.euo.2020.10.011 33199252

[39] Sanchez DF , Cañete S , Fernández‐Nestosa MJ , et al. HPV‐ and non‐HPV‐related subtypes of penile squamous cell carcinoma (SCC): morphological features and differential diagnosis according to the new WHO classification (2015). Semin Diagn Pathol. 2015;32:198‐221. 25701382 10.1053/j.semdp.2014.12.018

[40] Khalil MI , Kamel MH , Dhillon J , et al. What you need to know: updates in penile cancer staging. World J Urol. 2021;39(5):1413‐1419. 32572556 10.1007/s00345-020-03302-z

[41] De Felice F , Bird T , Michaelidou A , et al. Radical (chemo)radiotherapy in oropharyngeal squamous cell carcinoma: comparison of TNM 7th and 8th staging systems. Radiother Oncol. 2020;145:146‐153. 31981964 10.1016/j.radonc.2019.12.021

[42] Lont AP , Kroon BK , Horenblas S , et al. Presence of high‐risk human papillomavirus DNA in penile carcinoma predicts favorable outcome in survival. Int J Cancer. 2006;119:1078‐1081. 16570278 10.1002/ijc.21961

[43] Bezerra AL , Lopes A , Santiago GH , Ribeiro KC , Latorre MR , Villa LL . Human papillomavirus as a prognostic factor in carcinoma of the penis: analysis of 82 patients treated with amputation and bilateral lymphadenectomy. Cancer. 2001;91:2315‐2321. 11413520

[44] Necchi A , Pederzoli F , Bandini M , Spiess PE . Revolutionizing care for rare genitourinary tumours. Nat Rev Urol. 2021;18:69‐70. 33235393 10.1038/s41585-020-00402-8

